# Bone Metabolism in Patients Treated for Depression

**DOI:** 10.3390/ijerph17134756

**Published:** 2020-07-02

**Authors:** Elżbieta Skowrońska-Jóźwiak, Piotr Gałecki, Ewa Głowacka, Cezary Wojtyła, Przemysław Biliński, Andrzej Lewiński

**Affiliations:** 1 Department of Endocrinology and Metabolic Diseases, Medical University of Lodz, 93-338 Lodz, Poland; andrzej.lewinski@umed.lodz.pl; 2 Department of Endocrinology and Metabolic Diseases, Polish Mother’s Memorial Hospital—Research Institute, 93-338 Lodz, Poland; 3 Department of Adult Psychiatry, Medical University of Lodz, 91-229 Lodz, Poland; piotr.galecki@umed.lodz.pl; 4 Department of Laboratory Diagnostics, Polish Mother’s Memorial Hospital—Research Institute, 93-338 Lodz, Poland; ewa.biol@interia.pl; 5 Department of Oncological Gynecology and Obstetrics, Center of Postgraduate Medical Education, 00-416 Warsaw, Poland; czwo@op.pl; 6 International Prevention Research Institute—Collaborating Centre, State University of Applied Sciences, 62-800 Kalisz, Poland; 7 Faculty of Heath Sciences, State University of Applied Sciences, 62-800 Kalisz, Poland; bildom@gmail.com; 8 Copernicus Memorial Comprehensive Cancer Center and Traumatology, 93-513 Lodz, Poland

**Keywords:** depression, bone markers, osteocalcin, β-CTX, vitamin D

## Abstract

Background: Depression and osteoporosis are severe public health problems. There are conflicting findings regarding the influence of depression on bone metabolism. The aim of the presented study was to compare bone turnover markers and vitamin D levels between patients treated for depression and healthy controls. Patients and Methods: We determined a concentration of osteocalcin, carboxy-terminal telopeptide of type I collagen (β-CTX), 25-hydroxyvitamin D (25OHD) and 1,25(OH)_2_D_3_ in 99 patients, aged 46.9 ± 11 years, treated for depression, as well as in 45 healthy subjects. Depressive status was determined with the Hamilton Depression Scale (HDRS). Results: In patients treated for depression, we demonstrated significantly lower osteocalcin concentrations (*p* < 0.03) and higher concentration of β-CTX (result on the border of significance; *p* = 0.08). Those relationship were stronger in women. The level of 25OHD and 1,25(OH)_2_D_3_ did not differ significantly between the examined groups. We observed a negative correlation between the 25OHD and HDRS score after treatment in all patients treated for depression and in subgroups of women and subjects with recurrent depression. Conclusions: Our results indicate that depression is related to disturbances in bone metabolism, especially in women and patients with recurrent depression, suggesting its role in context of osteoporosis development.

## 1. Introduction

Osteoporosis is a severe public health problem. The essence of the disease is reduced bone strength, predisposing to an increased risk for fractures. As many as 9 million low-energy fractures are recorded worldwide every year [1,2,3]. According to the International Osteoporosis Foundation (IOF) data, over 200 million women in the world suffer from osteoporosis, the majority being above 60 years of age. In 2017, new fragility fractures in Europe were estimated at 2.7 million with an associated annual cost of €37.5 billion and a loss of 1.0 million quality-adjusted life years, with a projected increase to 3.3 million in 2030 [3]. Osteoporotic fractures are a major cause of morbidity and mortality in the population [2,3]. Data obtained in 2005 from the Polish Health Care Fund gives information about 17,625 hip fracture (mean incidence 224/100,000) [4]. A total of 289,230 hip fractures were observed in Poland in the years 2008–2015, with the incidence increasing from 192/100,000 in 2008 to 444/100,000 in 2015 [5]. The one-year mortality ranged between 30.45% and 32.8% for men and 26.2% and 28% for women [5]. In Europe, osteoporosis accounted for more disability and life years lost than rheumatoid arthritis, but less than osteoarthritis. With regard to neoplastic diseases, the burden of osteoporosis was greater than for all sites of cancer, with the exception of lung cancer [2]. A large number of risk factors for fractures have been identified: age, gender, postmenopausal status, body mass index, and smoking [6,7]. Identification of risk factors is important for early diagnosis and treatment, resulting in a significant public health benefit [7]. Recently, depression and its treatment were suspected as risk factors for osteoporosis development [8,9,10]. The problem is a significant public health issue since depression affects 98.7 million people globally, being a major cause of disability worldwide [11]. Depression has been associated with a low bone mineral density [8,9]. Moreover, the increased risk of fracture in patients with depression was demonstrated; 26% when the hazard risk was assessed (*n* = 309,862) and 39% when the risk ratio was determined (*n* = 64,975) [9]. Data on the influence of depression therapy on bone metabolism, especially with SSRIs (selective serotonin reuptake inhibitors), are conflicting [10,12]. There are many hypotheses associating depression with osteoporosis [13,14,15,16,17,18,19,20]. The first mechanism is similar to steroid-induced osteoporosis and affects the hypothalamic–pituitary–adrenal axis; in subjects suffering from depression elevated ACTH and cortisol levels have been demonstrated [13,14]. Moreover, in depressed individuals, glucocorticoid tissue hypersensitivity due to a greater prevalence of Bcl1 polymorphism has been observed [15]. The second mechanism is related to overproduction of inflammatory cytokines, such as interleukins IL-1β, IL-2, IL-6, and tumor necrosis factor-alpha, in patients with depression [16]. Other, probably coexisting factors to consider are the propensity to fall [17], low mobility [18], adoption of poor health behaviors (e.g., smoking and physical inactivity), and drugs, especially SSRIs [10,12,18]. Vitamin D deficiency seems to be an important factor, connecting osteoporosis with depression [8,9,10]. Vitamin D has a dual role in the human body, acting as a hormone and fat-soluble vitamin, regulating the expression of over 900 genes by binding to the vitamin D receptor (VDR) [19]. The presence of VDR receptors has been confirmed in more than 30 cell types in the human body, including nerve cells as well as in the glia of the cerebral cortex and hippocampus [20], locations relevant to depression [21]. The enzyme necessary for the hydroxylation of 25OHD to its active form, 1,25(OH)_2_D_3_, is present in the hypothalamus, cerebellum, and black substance (substantia nigra) [22]. Moreover, deficiency of vitamin D is a global problem [23], what also has been confirmed both in the general Polish population [24] and the population of Polish patients suffering from depression [25].

Bone metabolism has never been assessed in a population of Polish patients suffering from depression. The aim of the present study was to compare bone metabolism and vitamin D supply between patients treated for depression and the healthy controls.

## 2. Patients and Methods

The procedures used in the study were approved by Bioethics Committee of the Polish Mother’s Memorial Hospital—Research Institute (consent number: No.75/2015).

Patients with depression participating in the study were recruited from patients undergoing hospital treatment in the Department of Adult Psychiatry of the Medical University of Lodz.

The inclusion criteria for participation in the depression group were based on the diagnostic criteria of depressive episodes and recurrent depressive disorder according to International Classification of Diseases (ICD-10) (F32.0-7.32.2, F33.0-F33.8) [ICD-10]. Exclusion criteria for participation in the depression group were as follows: central nervous system injuries; history of inflammatory, autoimmune, and cancer diseases; abuse or addiction to psychoactive substances; lack of consent to participate in the study diseases; and/or treatments that affect bone turnover (except vitamin D supplementation—during hospitalization patients were taking 1000 IU of vitamin D daily.) The list of factors affecting bone metabolism consisted of a history of osteoporosis; thyroid diseases; rheumatoid arthritis; malabsorption; hypogonadism; undergoing treatment with the usage of substances, such as steroids, bisphosphonates, and denosumab; and abnormal result of any of the following laboratory tests: complete blood count, creatinine, calcium, phosphates, CRP (C-reactive protein), TSH (thyroid stimulating hormone), and testosterone. Patients with depression were divided into two subgroups—those with a first episode of depression and those with recurrent depression.

Healthy, non-depressed controls were recruited from the Outpatient Clinic of Osteoporosis Polish Mother’s Memorial Hospital—Research Institute, Lodz, after exclusion of osteoporosis.

Exclusion criteria to participate in the control group were a negative history of depression, osteoporosis, and diseases and/or treatments that affect bone turnover (corresponding to group of patients with depression). Nine subjects were excluded from the study due to a diagnosis of primary hyperthyroidism (*n* = 2), hypothyroidism (*n* = 2), renal failure (*n* = 1), hypocalcemia (*n* = 1), steroid treatment (*n* = 1), failed vitamin D assessment (*n* = 1), and lack of consent (*n* = 1). Qualification to participate in the study was random, based on non-returnable draw rules. Participation in the study did not affect the principles of treatment. Before proceeding with the study, each of the subjects signed an informed consent to participate in the study in accordance with the protocol.

The Hamilton Depression Rating Scale (HDRS) was used to assess the severity and dynamics of the symptoms of depression [26] on the admission to hospital (HDRS I) and after obtaining clinical improvement, on average after 8 weeks of treatment (HDRS II). We used the Polish version of the HDRS, validated by Wiglusz and et al. [27], containing 17 items, conducted by qualified personnel. HDRS-17 demonstrated the best psychometric properties for a cutoff score of 11 with a sensitivity of 100%, specificity of 89.3%, positive predictive value of 72.4%, and negative predictive value of 100% [27].

Regarding the biochemical measurements, osteocalcin (OC), a non-collagenous protein of the bone matrix, synthesized by osteoblasts, was chosen as a marker of bone formation. As a marker of bone resorption, we used the carboxy-terminal telopeptide of type I collagen (β-CTX) serum assay. OC, β-CTX, and vitamin D (25OHD) were determined by commercially available electrochemiluminescence immunoassays (ECLIA Cobas e601, Roche, Rotkreuz, Switzerland). The 1,25(OH)_2_D_3_ measurement was performed by automated direct immunoassay on the Liaison XL (DiaSorin S.p.A, Saluggia, Italy). The intra-assay and inter-assay CVs were less than 4.0%.

Statistics: Shapiro–Wilk’s test was used to assess the distribution of the variables. A one-way ANOVA was applied for statistical analysis for continuous variables with the subsequent use of a post-hoc test in order to assess differences between particular groups. In case of a non-normal distribution of data, Mann–Whitney’s test was used. Associations between HDRS and 25OHD were investigated with Spearman’s coefficient of correlation. Statistically significant differences were accepted when the *p* value was below 0.05. The analyses were carried out using STATISTICA 13 software (StatSoft, Kraków, Poland).

## 3. Results

We recruited 144 subjects to the present study; 99 patients treated for depression and 45 healthy subjects constituting the control group. Characteristics of the studied population are presented in Table 1. The compared groups do not differ significantly according to age, gender, BMI, and smoking (*p* > 0.1).

We demonstrated significantly lower osteocalcin concentrations in patients treated for depression when compared to the healthy controls—Table 2. We also found a higher concentration of βCTX in depression, however it not did reach the level of significance. The level of 25OHD and 1,25(OH)_2_D_3_ did not differ significantly between the studied groups—Table 2.

When women and men were analyzed separately, we observed a significantly higher β-CTX concentration in women with depression than in healthy women—Table 3. Similarly, we noticed a difference between the concentration of 25OHD and osteocalcin, but the results were on the level of significance. In contrast, in the group of men we did not find such relations (*p* = NS). Women and men with depression did not differ according to age (*p* = 0.42), duration of disease (*p* = 0.85), and HDRS I or HDRS II results (*p* = 0.4 and *p* = 0.99, respectively).

Mean concentration of 25OHD did not differ in the examined groups. The optimal concentration of 25OHD (>30 ng/mL) was found only in 4.3%, despite cholecalciferol supplementation. Deficiency of vitamin D was frequently observed in the studied population, both in the depression group and the control group. We found significant correlation (*p* < 0.05) between the 25OHD levels and HRDS II score in all studied patients with depression (rs = −0.33,), in all women with depression (rs = −0.56), in subjects with recurrent depression (rs = −0.39), and in women with recurrent depression (rs = −0.45). Moreover, in all mentioned groups, 25OHD was significantly higher in the groups of patients with an HDRS II score below 7 points compared to patients with an HDRS II score higher or equal to 7 points (*p* < 0.03).

## 4. Discussion

We demonstrated that markers of bone turnover in patients treated for depression took values characteristic of accelerated bone loss (lower osteocalcin and elevated β-CTX), which might lead to osteoporosis development in that group of patients. That relationship was stronger in women with depression compared to the healthy controls. These results are similar to those observed by other researchers [14,18,28,29,30,31], who also studied young women with major depression. Generally, women are most likely to develop osteoporosis [1,2,3]. However, this statement applies rather to postmenopausal women. Interestingly, in our study young women in the premenopausal period were at risk. The data we collected did not confirm increased bone turnover in men, while data from the literature concerning males are divergent, both according to age and drug use [29,31].

A lot of data on vitamin D deficiency on the course of depression has been published [25,32,33]. According to [32] (*n* = 5607 subjects) and [33] (*n* = 1602 subjects), vitamin D deficiency can be a significant cause of late-life depression in older people, especially among men over 65 years of age. In addition, in a 10-year longitudinal study, the relationship between the 25OHD level and the effectiveness of cognitive processes dependent on the prefrontal cortex was confirmed [34]. This is further supported by findings showing effects of 1,25-OH_2_D_3_ on the synthesis of nerve growth factor (NGF). 1,25-Dihydroxyvitamin D3 is a potent inducer of nerve growth factor synthesis [35]. The synthesis of tryptophan is also transcriptionally activated by vitamin D; thus, low 25OHD levels may accordingly cause low levels of serotonin [36].

Surprisingly, in our study, unlike previous data [25,37], neither 25OHD nor 1,25(OH)_2_D_3_ levels in patients with depression were different from the healthy controls. Even paradoxically, in women with depression, the 25OHD concentration was higher than in the controls. It was related to supplementation of vitamin D by patients with depression. This supplementation, while conducted irregularly without dose titration, led to increasing vitamin D concentration and achieving the level similar to the control group not taking vitamin D. However, in all studied subjects, the 25OHD levels were lower than the optimal according to recommendations of the Endocrine Society [23]. Since we observed the negative correlation between 25OHD and the Hamilton score after treatment in all depressed patients, particularly in women and in patients with recurrent depression, we advocate for more careful supplementation of vitamin D in that group of patients. We are aware of conflicting results of vitamin D supplementation in depression [37,38,39,40], so we treat this supplementation rather as a necessary element of osteoporosis prophylactics. Nevertheless, it is noteworthy that the beneficial effects of vitamin D supplementation among those with a replete vitamin D status were suggested [37,40].

We are aware of the limitations of our study, which include a lack data according diet habits—in particular calcium and protein intake, alcohol consumption, and physical activity. There is no doubt that a relatively strong association exists between osteoporosis and the mentioned factors, but we would like to emphasize that the survey methods of their assessment still do not fulfill the criteria of being valid, reliable, and practical, which was shown in relation to physical activity [41] and especially to alcohol consumption [42]. Other limitations are a lack of densitometry results and a small number of studied patients; however, the number is comparable with other studies [18,28]. Strengths of the study included an investigation carried out on a large group of well-defined patients with depression, seeming to be representative enough for the Polish population.

In conclusion, our study denotes a relationship between depression and bone metabolism, particularly in women and in patients with recurrent depression, likely predisposing osteoporosis development. However, future trials are needed, targeting depressed patients for developing an optimal model of osteoporosis prophylactics in these patients.

## Figures and Tables

**Table 1:** Characteristics of the studied group presented as the mean ± SD. HDRS I (Hamilton Depression Rating Scale score on admission); HDRS II (Hamilton Depression Rating Scale score after obtaining clinical improvement).

	Controls *n* = 45	All Patients with Depression *n* = 99	The First Episode of Depression *n* = 31	Recurrent Depression *n* = 68
Age (years)	45.8 ± 10.8	46.9 ± 11	43.3 ± 12.5	49.5 ± 9.9
Women/men	24/21	55/42	17/14	38/27
HDRS I	-	22.9 ± 7.2	21.9 ± 7.1	23.4 ± 7.1
HDRS II	-	6.6 ± 4.1	5.4 ± 4.0	6.2 ± 3.9
Number of depressive episodes	-	5.9 ± 3.9	-	6.8 ± 3.5
Duration of disease (years)	-	6.9 ± 5.5	1.9 ± 1.8	9.1± 8.4

**Table 2:** Vitamin D and bone markers in patients with depression and the healthy controls. Data presented as the mean ± SD; 25OHD—25-hydroxyvitamin D; 1,25(OH)_2_D_3_—1,25-dihydroxyvitamin D_3_; β-CTX—carboxy-terminal telopeptide of type I collagen; *p*-level of significance.

	Reference Range	Depression	Controls	*p*
25OHD (ng/mL)	Optimal > 30 Insufficiency 20–30 Deficiency < 20	18.84 ± 7.09	17.56 ± 6.6	0.32
1,25(OH)_2_D_3_ (ng/L)	29–83.6	51.33 ± 15.37	55.95 ± 7.07	0.37
β-CTX (ng/dL)	130–710	507 ± 320	389 ± 158.8	0.08
Osteocalcin (ng/mL)	15–46	19.17 ± 7.86	22.88 ± 9.44	0.03

**Table 3:** Vitamin D and bone markers in women with depression and healthy controls. Data presented as the mean ± SD; 25OHD—25-hydroxyvitamin D; 1,25(OH)_2_D_3_—1,25-dihydroxyvitamin D_3_; β-CTX—carboxy-terminal telopeptide of type I collagen; *p*-value level of significance.

	Women with Depression	Healthy Women	*p*
25OHD (ng/mL)	19.34 ± 6.31	16.57 ± 6.74	0.055
1,25(OH)_2_D_3_ (ng/L)	51.67 ± 15.32	50.33 ± 18.31	0.9
β-CTX (ng/dL)	521.08 ± 290.34	314.59 ± 115.78	0.01
Osteocalcin (ng/mL)	19.37 ± 7.34	23.17 ± 10.22	0.059
